# First report of *Toxoplasma gondii* in camels (*Camelus dromedarius*) in Ethiopia: bioassay and seroepidemiological investigation

**DOI:** 10.1186/s12917-014-0222-7

**Published:** 2014-09-30

**Authors:** Endrias Zewdu Gebremedhin, Hasen Awel Yunus, Gebregergs Tesfamaryam, Tesfaye Sisay Tessema, Fufa Dawo, Getachew Terefe, Vincenzo Di Marco, Maria Vitale

**Affiliations:** Department of Veterinary Laboratory Technology, Ambo University, Faculty of Agriculture and Veterinary Sciences, P.O. Box 19, Ambo, Ethiopia; Department of Animal Sciences, Faculty of Agriculture, Mizan Tepi University, P.O. Box 260, Mizan, Ethiopia; College of Veterinary Medicine, Jigjiga University, P.O. Box 307, Jijiga, Ethiopia; Institute of Biotechnology, College of Natural and computational Sciences, Addis Ababa University, P.O. Box 1176, Addis Ababa, Ethiopia; College of Veterinary Medicine and Agriculture, Addis Ababa University, P.O. Box 34, Debre Zeit, Ethiopia; Italian National Reference Centre for Toxoplasmosis at Istituto Zooprofilattico Sperimentale della Sicilia A. Mirri, Palermo, Italy

**Keywords:** *T. gondii*, Camel, DAT, ELISA, Seroepidemiology, Bioassay, Ethiopia

## Abstract

**Background:**

Toxoplasmosis is a major public health concern in many countries of the world. A cross-sectional and follow up experimental study designs were used for seroepidemiological and bioassay studies, respectively from November 2012 to April 2013. The objectives were to estimate the seroprevalence of *T. gondii* infection, to assess risk factors and to isolate the parasite from camels in the Fentale district, Ethiopia. A direct agglutination test (DAT) and indirect enzyme linked immunosorbent assay (ELISA) kits were used to test camel sera. Hearts and tongues (each 25 g) from 31 seropositive camels were bioassayed in mice. Associations between seroprevalence and potential risk factors (collected using a questionnaire survey) were analyzed using logistic regression.

**Results:**

An overall *T. gondii* prevalence of 49.62% (220/455) by DAT and 40.49% (179/451) by indirect ELISA test were detected. Herd level seroprevalence of 96.77% (30/31) (95% CI: 83.30– 99.92) by DAT was recorded and it was significantly higher in areas where wild felids are present (*P* = 0.038). Multivariable logistic regression showed that the likelihood of acquiring *T. gondii* infection was significantly higher in camels in the Ilala pastoral association [PA] (82.26%) (Adjusted Odds ratio [aOR] = 10.8; *P* < 0.001) than camels in the Galcha PA (31.43%), in camels of ≥ 8 years old (56.52%; aOR = 1.88; *P* = 0,033) than camels of ≤ 4 years old (34.26%) and in areas where domestic cats are present (aOR = 4.16; *P* = 0.006). All camel owners were uneducated, handle aborted fetus with bare hands, and drink raw camel milk. DAT and ELISA tests had moderate agreement (Kappa = 0.41). Viable *T. gondii* were isolated from 16.13% (5/31) of DAT positive camels. One DAT positive but ELISA negative camel sample gave a cyst positive result.

**Conclusions:**

*T. gondii* infection of camels in the study district is widespread. Age, presence of domestic cats and study PA are independent predictors of *T. gondii* seropositivity. Isolation of viable parasites from edible tissues of camels and the very poor knowledge of pastoralists about toxoplasmosis suggest the need for prevention of toxoplasmosis through bio-security measures, education and further investigation to unravel the impact of camel toxoplasmosis deserves consideration.

**Electronic supplementary material:**

The online version of this article (doi:10.1186/s12917-014-0222-7) contains supplementary material, which is available to authorized users.

## Background

A population over 2.3 million camels is estimated in Ethiopia [1], which is considered the third largest camel populated country in the world. All camels in Ethiopia are owned by pastoralists [2]. Camels play a central role in the livelihood of pastoralists through provision of milk, meat, and draught power and they also determine their wealth and social status [3]. Despite their social, ecological and economic importance in Ethiopia, until recently they were neglected by researchers and development planners and as a result little is known about productivity and health problems in camels compared to other livestock [4,5].

Camels suffer from various parasitic diseases, including toxoplasmosis, which have public health and economic importance [6]. Toxoplasmosis is caused by an intracellular Apicomplexa protozoan, *T. gondii,* found worldwide and in an exceptionally broad host range on earth [7]. Camels acquire *T. gondii* infection through ingestion or inhalation of sporulated oocysts that are shed by cats or wild felids in the environment [8]*.* The prevalence of *T. gondii* infection in camels varies widely depending on the localities of the world [9], ranging from 3.12% in Iran [10] to 90.90% in Turkey [11].

*T. gondii* infects one-third of the world human population. The infection can be life threatening during pregnancy and in immunocompromised individuals [12,13]. The disease is a major public health concern in many countries of the world [14]. In USA, it is the third leading cause of death from foodborne diseases [12].

Humans become infected with *T. gondii* mostly by ingesting raw or undercooked meat of infected animals or by ingesting food or water contaminated with oocysts [13] and the parasite can be transmitted to the fetus by the passage of tachyzoites through the placenta [15]. The presence of *T. gondii* in milk [10] and edible tissues [16] of carrier camels indicate the possibility of transmission to humans; particularly in pastoral communities in which raw milk, and to some extent raw meat, can be frequently consumed [17]. Recent reports from Ethiopia indicate high seroprevalence of toxoplasmosis in women of child-bearing age ranging from 70.29 to 87.4% [18-20].

Studies done in different parts of Ethiopia over the last 24 years indicated high seroprevalence of *T. gondii* infection in sheep [21-23], goats [21,22,24,25], cattle [21], chicken [26] and cats [27]. However, there is no single report of *T. gondii* infection in Ethiopian camels so far. Although DAT and ELISA tests have been widely used for detection of *T. gondii* infection in epidemiological studies, there are no reports on the comparative evaluation of the diagnostic capability of these diagnostic tests in naturally infected camels. This report compares serodiagnostic methods of *T. gondii* infection in camels using commercial kits (DAT and ELISA), and isolation of the parasite from edible camel tissues, to estimate the seroprevalence of toxoplasmosis in camels. The role of the identified risk factors on the parasite transmission is also discussed.

## Methods

### Study area and study animals

**Figure 1:**
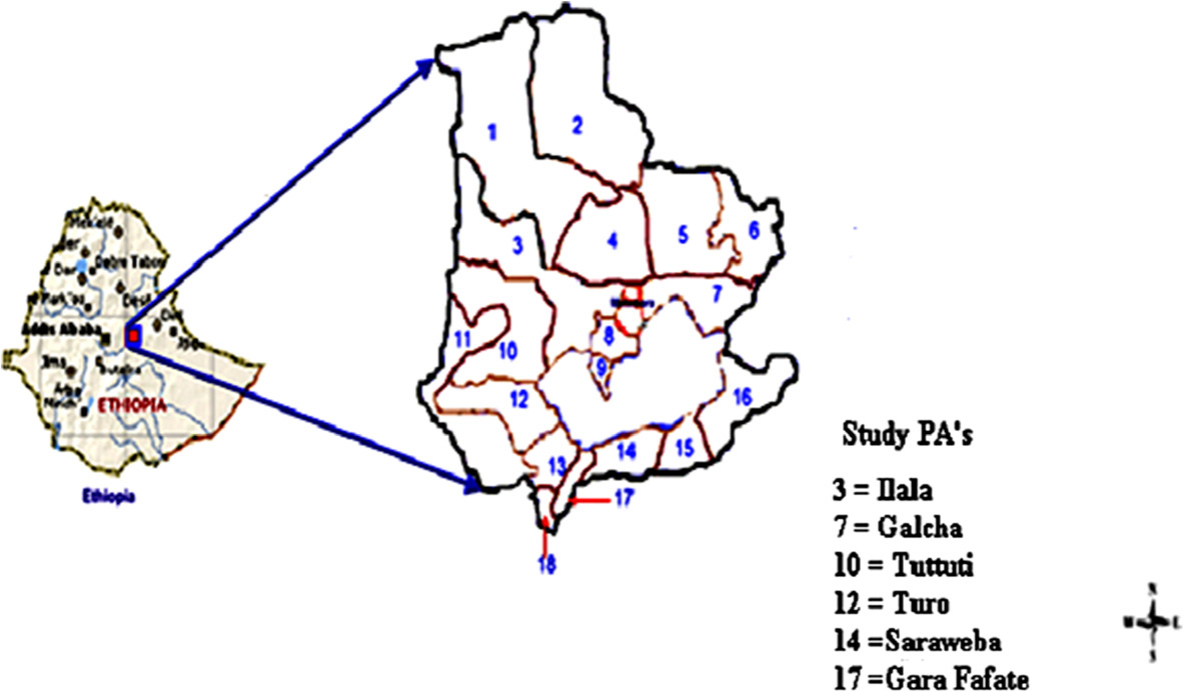
**Map of the Fentale district showing sampled pastoral associations (PAs) (adapted from** https://www.google.com.et/search?q=Ethiopia+map&btnG=&tbm=isch&gws_rd=ssl **)**.

The study was conducted in the Fentale district (39.93°E to 39°56’0”E longitude and 8.975^o^ N to 8.58’30” N latitude) which is located in the East Shewa Zone of Oromia Regional State, Ethiopia (Figure 1) at 190 Kms East of Addis Ababa. It lies at an altitude range of 955-2007 meters above sea level, and has an annual rainfall ranging from 560 millimeter (mm) to 630 mm and mean temperature ranging from 29°C to 38°C. The vegetation comprises mix of acacia trees, bushes and shrubs that are common to the lowland areas of Ethiopia. With its arid and semi-arid climate, pastoral and agro-pastoral production systems predominate in the area. The district is estimated to possess 48, 078 camels found in eighteen pastoral associations (PAs). Pastoral and agro-pastoral production systems are practiced in 11 and 7 PAs, respectively [28].

The study population consisted of camels of different age groups in selected PAs of the district managed under the two production systems. The camels of both sex groups were classified by age into three categories (≤4 years, 4-8 years and > 8 years) [29]. Ages of camels were recorded based on information from the owners and also estimated according to their dentations [30].

### Study design, sample size determination and sampling method

Cross-sectional and follow up experimental study designs were used for seroepidemiological and bioassay studies, respectively from November 2012 to April 2013. Out of 18 PAs, a total of 6 PAs (pastoral = 3, agro-pastoral = 3) were chosen conveniently based on accessibility of the PAs. Camel herds in each of the selected PAs were stratified by herd size into three: small (1-9), medium (10-19) and large (≥20) camels [31]. Out of 58 camel herds found in the selected study PAs (n = 6), 31 herds were randomly selected using the lottery method. Then, all camels in selected herds were used to collect blood samples. Since there has been no previous study on camel toxoplasmosis in Ethiopia, 50% expected prevalence (P) and 95% confidence interval (Z = 1.96) with a 5% desired absolute precision (d) was considered to arrive at the required sample size (n = 384) for the seroepidemiological study using the formula: n = P (1-P) Z^2^ /d^2^) [32]. Additionally, purposive sampling of blood from 70 camels was considered for sample selection by serology in order to use their heart and tongue for bioassay.

### Sample collection and transportation

Blood samples for serum separation were collected from the jugular vein of apparently healthy camels using labeled plain vacutainer tubes following proper physical restraining. For the bioassay, heart, tongue and blood sample of purposively selected 70 camels, originating from Fentale district but slaughtered at Akaki abattoir near Addis Ababa, were collected. The collected samples were kept in an icebox containing ice packs and immediately transported to the Ethio-Belgium post-graduate research laboratory, College of Veterinary Medicine and Agriculture, Debre Zeit, Ethiopia for serological testing and isolation of *T. gondii*. Sera were separated by centrifugation at 3200 rpm for 10 minutes, decanted into cryovials and stored at -20°C until used for serological assay. Heart and tongue samples were kept at + 4°C for 1-3 days until serological results were known.

### Questionnaire survey

A close-ended questionnaire was designed and self-administered to collect data about the potential risk factors for camels to acquire *T. gondii* infection. Awareness of camel owners about toxoplasmosis including the habits of consumption of raw camel meat and milk was also documented. The questions in the questionnaire included sex (male, female), age (≤4 years, 4-8 years and > 8 years), production system (pastoral/agropastoral), presence of cats (yes/no), presence of wild felids (yes/no), source of drinking water (tap, stagnant: [lake, pond and well], river, mixed [river and stagnant]) and herd size (small:1-9, medium:10-19, large: ≥ 20 camels) [31]. Information about history of abortion (yes/no), way of handling aborted fetus (wearing gloves, without wearing glove, washing hand after handling, without washing hand after handling) and method of disposal of aborted fetus (giving to cat, throwing along the way, burying) were recorded. Moreover, camel owners consumption habits of camel meat (cooking, without cooking, both) and raw milk consumption (yes/no), educational level (uneducated, primary school, secondary school, tertiary/university) and awareness about: toxoplasmosis (yes/no), role of cat to transmit diseases to owner (yes/no), role of cat to transmit diseases to camel (yes/no) and camel migration (yes/no) and routes of migration were also gathered.

### Laboratory investigation

#### Direct agglutination test (DAT)

All sera samples collected were examined for the presence of antibodies (IgG) against *T. gondii* by the direct agglutination test (Toxo screen DA, biomerieux®, France) following the protocol of the manufacturer. The kit was claimed by the manufacturer to have sensitivity and specificity of 96.22% and 98.80%, respectively. Sera were assayed at a screening dilution of 1:40 and 1:4000. A titer of 1:40 or 1:4000 or both was considered indicative of *T. gondii* exposure. Sedimentation of antigen at the bottom of the well and clear agglutination above half of the well at either dilution were recorded as negative and positive results respectively. DAT positive samples were titrated to know the endpoint titer. Positive and negative controls were included in each test.

#### Indirect enzyme linked immunosorbent assay (ELISA)

A total of 451 (381 from field and 70 from abattoir) sera were tested for *T. gondii* IgG antibodies by the indirect ELISA kit (ID VET Innovative Diagnostic, ID Screen®, Montpellier, France) following the manufacturer’s instructions. The wells of the kit are coated with *T. gondii* P30 (SAG1) surface antigen. The kit uses non-species specific protein conjugate that is used in several animal species. For interpretation of the result S/P% was calculated as: S/P% = (optical density [OD] 450 value of the sample – OD 450 value of the negative control)/(mean OD 450 value of the positive control – OD 450 value of the negative control) × 100. Any sample with S/P% ≤ 40%, 40% - 50% and ≥ 50% were considered as negative, doubtful and positive, respectively. With this cut-off the manufacturer claims the test to have more than 99% specificity and sensitivity. The test was considered valid if the mean OD value of the positive control (ODPC) is greater than 0.350 (ODPC > 0.350) and the ratio of the mean OD value of the positive and negative controls (ODPC and ODNC) is greater than 3.5 (ODPC/ODNC > 3.5). The doubtful sera were retested.

#### Bioassay

Heart and tongue tissue samples (pooled weight 50 g) from DAT positive camels (n = 31) slaughtered at Akaki abattoir, were processed for isolation of *T. gondii* as described previously [13]. Briefly, each sample was cut into small pieces, homogenized in a blender for 30 seconds, followed by soaking in 125 ml of saline solution for another 30 seconds (0.14 M sodium chloride). After homogenization 250 ml of pepsin solution (Merck KG.A, Darmstadt, Germany) was added (pH = 1.1–1.2) and incubated at 37°C for 1 h. The homogenate was filtered through two layers of gauze and centrifuged at 3200 rpm for 10 minutes. The supernatant was discarded and to the sediment 15 to 20 ml of 1.2% sodium bicarbonate (pH = 8.3) was added and centrifuged at 3200 rpm for 10 min. The supernatant was discarded and the sediment was re-suspended in 5 to 10 ml of antibiotic saline solution (1000 U/ml penicillin and 100 μg of streptomycin/ml of saline solution). The suspension was inoculated intraperitoneally (1 ml per mouse) into five apparently healthy *T. gondii* seronegative female Swiss-Albino mice (National Veterinary Institute, Debre Zeit) per sample (weighting 25–35 g) as described by Dubey [13]. Bioassays were performed within 1-3 days of the slaughter of the camels. Separate knives, cutting boards, gloves, plastic cups/falcon tubes, and plastic bags for blending and pepsin digestion were used for each sample to avoid contamination among samples. Tissue homogenate kept in 6 falcon tubes (50 ml) were centrifuged in 2 steps; the first and second batch in 4 and 2 floating buckets of the centrifuge. Approximately 110-140 minutes elapsed from the time the tissue was homogenized in saline to the time the material was pulled into syringes for mouse inoculation. The inoculated mice were observed daily for illness for 60 days and information on number of survivors, dead, day of death, symptoms, and weight (initial and final) were recorded. The mice were fed with pelleted feed and municipal chlorinated water ad-libitum. *T. gondii* isolate was considered as virulent if 100% mortality of mouse was observed within four weeks of infection [33].

Surviving mice were bled on day 60 after anesthetizing with di-ethyl ether (Biolab laboratories ltd, Israel). Brains of all mice were removed by sagittal dissection and then examined for tissue cysts. The inoculated mice were considered infected with *T. gondii* when tissue cysts were found in brain tissues and/or the sera of mice react positively for the commercial DAT. The brain was homogenized in 1 ml phosphate buffer saline (PBS) by using a mortar and pestle. The numbers of cysts in five aliquots of each 10 μl were counted under microscope with a 10 × and 40 × objective lens and summed. The total number of cysts in the brain of each mouse was determined by multiplying the number of cysts in the 50 μl sample examined with the dilution factor [34].

### Data management and analyses

The data generated from field and laboratory investigations were entered and coded using Microsoft Excel® 2007 and analyzed using STATA version 11.0 for Windows (Stata Corp. College Station, TX, USA). Descriptive statistic was used to summarize the data. The information from the questionnaire survey was used to define explanatory variables to be tested in a logistic regression model, for any assessment of association between the serological status of the camels (dependent variable) and the risk factors (independent variables). Initially, all risk factors were individually screened by cross-tabulation for association with the likelihood of *T. gondii* infection using Chi-squared analysis. Non-collinear variables, with *P*-value < 0.25 in the univariable analysis, were entered into the multivariable logistic regression model. During the analyses the clustering nature of the outcome within a herd was considered by including herd as a clustering variable. This enabled us to use a clustered sandwich estimator i.e., robust standard error rather than the standard error of the parameters estimated using maximum likelihood method. The results of the logistic regression for each variable were expressed as *P*-value and odds ratio (OR) with 95% confidence intervals to measure the strength of associations. The seroprevalence results of DAT and ELISA tests were compared using the Chi-squared test and their concordance was determined by calculating the Kappa index. Using DAT as a reference test, the diagnostic sensitivity (Se), specificity (Sp), positive predictive value (PPV) and negative predictive value (NPV) of ELISA were calculated and interpreted following the recommendations of Dohoo et al. [35]. A student t-test was employed to assess the mean weight difference between seropositive and seronegative mice. Differences were considered significant when *P* ≤ 0.05.

### Ethical considerations

This research project was approved by the animal ethical committee of the College of Veterinary Medicine and Agriculture, Addis Ababa University. All efforts were made to minimize animal suffering during the course of the study. Informed written consents were obtained from all camel owners who participated in the study.

## Results

### Animal level seroprevalence

**Figure 2:**
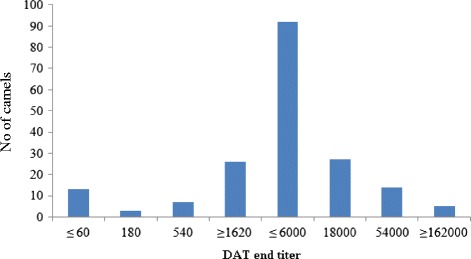
**DAT end titer of seropositive camels (n = 183).**

An overall *T. gondii* prevalence of 49.62% (220/455) by DAT and 40.49% (179/451) by indirect ELISA test were detected. Out of the 218 DAT positive sera, 16 (7.34%) were negative at 1:40 dilution but positive at 1:4000 dilution. The serum samples collected from the abattoir were excluded from risk factor assessment because of missing information for most variables. A statistically significant difference in seroprevalence was observed between studied PAs with the highest and lowest seroprevalence recorded from Ilala (82.26%) and Galcha (31.43%) PAs, respectively (*P* = 0.0001). The seroprevalence was also significantly different between age groups (*P* = 0.002), herd sizes (*P* = 0.011) and history of abortion (*P* = 0.003). However, there was no statistically significant difference in seroprevalence between males and females (*P* = 0.757) and pastoral and agro-pastoral production systems (*P* = 0.683; Table 1). Using ELISA, the PA (*P* = 0.003) and history of abortion (*P* = 0.038) were significantly associated with seropositivity (see Additional file 1: Table S1). Of the seropositive samples 73.8% (138/187) had DAT end titer of ≥ 6000. The DAT end titer of the seropositive camels (n = 187) is shown in Figure 2.

**Table 1:** ***T. gondii*** **seroprevalence in camels of the Fentale district, stratified by explanatory variables as detected by DAT**

**Variable**	**Categories**	**No. tested**	**No. positive**	**% seroprevalence**	**Chi-square**	***P*** **-value**
PA	Galcha	35	11	31.43	38.8441	<0.**001**
	Tuttuti	211	86	40.76		
	Saraweba	38	17	44.74		
	Garafafate	10	5	50.00		
	Turo	29	17	58.62		
	Ilala	62	51	82.26		
	Total	385	187	48.34		
Sex	Female	209	100	47.85	0.0961	0.757
	Male	176	87	49.43		
Age	≤4 yrs	108	37	34.26	12.6190	0.**002**
	4-8 yrs	185	98	52.97		
	>8 yrs	92	52	56.52		
Ps	Pastoral	308	148	48.05	0.1664	0.683
	Agro-pastoral	77	39	50.65		
Hs	Small	85	39	45.88	9.0675	0.**011**
	Medium	173	98	56.65		
	Large	127	50	39.37		
Abortion	No	198	90	45.45	8.6284	0.**003**
	Yes	11	10	90.91		
Stillbirth	No	207	98	47.34	2.2011	0.138
	Yes	2	2	100.00		
NNM	No	1	0	0.00	0.9218	0.337
	Yes	208	100	48.08		

PA = pastoral association, Hs = herd size, Ps = production system, NNM = neonatal mortality, P-values of statistically significant variables were highlighted in bold; No. = number.

### Herd level seroprevalence

Out of 31 camel herds examined, 30 (96.77%) (95% CI: 83.30– 99.92) and 29 (93.55%) (95% CI: 78.58– 99.21) herds were seropositive by the DAT and ELISA test, respectively (i.e., contain at least one seropositive camel). Using DAT, herd level seroprevalence of *T. gondii* infection was not significantly associated with PAs (*P* = 0.372), production system (*P* = 0.170), herd size (*P* = 0.576), history of abortion (*P* = 0.549) and presence of domestic cats (*P* = 0.549). However, there was significant association between herd seroprevalence and presence of wild felids (*P* = 0.038; Table 2). Using ELISA, herd level seroprevalence was not associated with the studied variables except neonatal mortality (see Additional file 1: Table S2).

**Table 2:** **Herd level seroprevalence of** ***T. gondii*** **infection in camels of the Fentale district as detected by DAT**

**Variable**	**Categories**	**No. tested**	**No. positive**	**% seroprevalence**	**Chi-square**	***P*** **-value**
PA	Galcha	4	4	100	5.3733	0.372
	Tuttuti	11	11	100		
	Saraweba	5	4	80		
	Garafafate	2	2	100		
	Turo	4	4	100		
	Ilala	5	5	100		
Ps	Pastoral	20	20	100	1.8788	0.170
	Agro-pastoral	11	10	90.91		
Hs	Small	15	14	93.33	1.022	0.576
	Medium	11	11	100		
	Large	5	5	100		
Abortion	Yes	8	8	100	0.3594	0.549
	No	23	21	95.65		
Stillbirth	Yes	3	3	100	0.1107	0.739
	No	28	27	96.43		
NNM	Yes	2	2	100	0.0713	0.790
	No	29	28	96.55		
Cat	Yes	8	8	100	0.3594	0.549
	No	23	22	95.65		
Wf	Yes	25	25	100	4.3056	0.**038**
	No	6	5	83.33		
River	Yes	29	29	100	-	-

PA = pastoral association, Hs = herd size, Ps = production system, Wf = presence of wild felids, NNM = neonatal mortality, No. = number.

### Risk factors for T. gondii seropositivity

#### Univariable logistic regression analysis

Epidemiological risk factors for acquiring *T. gondii* between DAT seroreactive and nonreactive camels were compared using univariable logistic regression analysis. During the statistical analysis the first level/category (with lowest seroprevalence) of each independent variable was used as the reference category. The results showed that sex, herd size and production system had no significant association with *T. gondii* seropositivity of the camels (*P* > 0.05) while study area, age, history of abortion, presence of domestic cats and presence of wild felids were found to be significantly associated with *T. gondii* infection (*P* < 0.05) (Table 3).

**Table 3:** **Animal level Logistic regression analysis of predictors of** ***T. gondii*** **infection in Camels as detected by DAT**

**Variable**	**Category**	**No. of positive/tested (%)**	**Univariable**		**Multivariable**	
			**OR (95% CI)**	***P***	**OR (95% CI)**	***P***
PA	Galcha	11/35 (31.43)	1		1	-
	Tuttuti	86/211 (40.75)	1.5 (0.58-3.88)	0.402	1.55 (0.60-3.99)	0.367
	Saraweba	17/38 (44.74)	1.77 (0.45-6.97)	0.417	1.5 (0.63-3.58)	0.357
	Garafafate	5/10 (50)	2.18 (0.87-5.45)	0.095	0.6 3 (0.17-2.33)	0.487
	Turo	17/29 (58.62)	3.1 (.77-12.34)	0.110	1.06 (.23-4.94)	0.940
	Ilala	51/62 (82.26)	10.11 (4.3-24.21)	<0.**001**	10.8 (4.83-24.17)	<0.**001**
Sex	Female	100/209 (47.85)	1			
	Male	87/176 (49.43)	1.06 (0.70-1.62)	0.767		
Age	≤4 yrs	37/108 (34.26)	1		1	-
	4-8 yrs	98/185 (52.97)	2.16 (1.28-3.64)	**0.004**	1.73 (0.99-3.02)	0.053
	>8 yrs	52/92( 56.52)	2.49 (1.34-4.65)	**0.004**	1.88 (1.05-3.37)	0.**033**
Ps^1^	Pastoral	148/308 (48.05)	1			
	Agro-pastoral	39/77 (50.65)	1.12 (0.46-2.64)	0.814		
Cat	No	173/305 (43.28)	1		1	-
	Yes	55/80 (68.75)	2.88 (1.28-6.47)	**0.010**	4.16 (1.49-11.61)	0.**006**
Wf^2^	No	18/80 (22.5)	1		1	-
	Yes	169/305 (55.41)	4.28 (2.40-7.63)	**<0.001**	2.04 (0.86-4.83)	0.104
Abortion	No	90/198 (45.45)	1		1	-
	Yes	10/11 (90.91)	12.0 (1.25-114.96)	**0.031**	9.23 (0.8-107.05)	0.075
Hs^3^	Small	39/85 (45.88)	1			
	Medium	98/173 (56.65)	1.54 (0.59-4.01)	0.376		
	Large	50/127 (39.37)	0.76 (0.26-2.29)	0.633		

^1^Production system, ^2^Wild felids, ^3^Herd size.

#### Multivariable logistic regression analysis

Stillbirth and neonatal mortality were not included in the multivariable model due to collinearity with abortion. Five variables (study area [PAs], age, abortion, exposure to domestic and wild cats) that presented *P*-value < 0.25 in the univariable analysis, were subjected to the multivariable regression model. The analysis showed a significant association between *T. gondii* seropositivity and study area (PAs) (*P* = 0.0001), age (*P* = 0.033) and the presence of domestic cats (*P* = 0.006; Table 3). The results of the logistic regression analysis based on the results of the ELISA were presented as Additional file 1: Table S3).

### Camel owners food habit and awareness about toxoplasmosis

All (100%) camel owners who responded to the questionnaire survey were uneducated pastoralists, had no awareness about *T. gondii,* toxoplasmosis and its transmission to humans through raw meat and raw milk consumption. Camel meat consumption habit showed that 67.7% consume cooked meat and the rest (32.3%) consume either cooked or uncooked meat. All respondents (100%) consume raw camel milk, handle aborted fetus with bare hands (no protection) and don’t wash their hands after handling aborted fetus. Furthermore, none of the camel owners bury or burn aborted materials; rather they simply dispose of by throwing it in the field.

### Comparison of seroprevalence results of DAT and ELISA tests

Overall, the 451 serum samples were tested by both DAT and ELISA kits for comparison. A total agreement between the two tests was obtained on 132 positive samples (29.27%) and 186 (41.24%) negative samples. But 86 sera were positive only by DAT test and 47 sera were positive only by ELISA test. Comparison of the two test results indicated that seropositivity differed significantly between the two kits (*χ*2 = 76.714, *P* < 0.001). However, Kappa analysis demonstrated that the two tests had a moderate agreement in detecting *T. gondii* infection in camels (70.51% agreement, Kappa = 0.41; *P* < 0.0001). By considering DAT as a reference test, the sensitivity, specificity, and positive and negative predictive values of ELISA was calculated as 60.55%, 79.83%, and 73.74% and 68.38%, respectively.

### Bioassay

Of the 70 camel sera from the abattoir, 33 (47.1%) (95% CI: 35.09-59.45) and 29 (41.43%) (95% CI: 29.77-53.83) were IgG seropositive using DAT and ELISA, respectively. Viable *T. gondii* cysts were isolated from 5 of 31 DAT seropositive bioassayed camels (16.13%). Out of the 31 DAT positive sera, 22 were positive for ELISA and one ELISA negative camel sample gave a cyst positive result. For two seropositive camel samples, isolation of the parasite was not possible because of the death of all of the inoculated mice within a day of inoculation. On day 60 post-inoculation, out of 108 mice that survived, 22 (20.4%) were seropositive for *T. gondii* infection (i.e., out of 31 camel samples, 10 (32.3%) gave a seropositive result on mice). Viable cysts were isolated in mice from 5 tissue samples from the camels (on 5 seropositive and 1 seronegative mice) (Table 4). It was not possible to detect cysts from 17 of the seropositive mice. Eleven out of 108 survived mice showed loss of weight (10.2%), of which 9 and 2 mice were seropositive and seronegative, respectively, i.e., 40.9% (9/22) of seropositive and 2.3% (2/86) of seronegative mice showed loss of weight. The mean weight change between seropositive (infected, mean = 0.25 g ± 6.58) and seronegative (non-infected, mean = 5.55 g ± 3.41) mice was significantly different (*P* < 0.0001). The mean cyst count per mouse brain was 46.5 (range: 28 – 88). One mouse that showed neurological signs (hind quarter paralysis, torticollis) and tachypnoea on day 20 post-inoculation became cyst negative but seropositive.

**Table 4:** **Isolation of** ***T. gondii*** **from naturally infected seropositive camels of East Shewa Zones, Central Ethiopia**

**Camel no.**	**Sex**	**Age group**	**DAT screening**	**Mice with cyst/examined (n)**	**Mice seropositive/examined (n)**	**Days of mice death PI (no. of mice dead)**
H1	M	≥8 Yrs	1/40	0/5	0/5	
H2	M	≥8 Yrs	1/40 & 1/4000	0/5	0/5	
H4	M	4-8 Yrs	1/40 & 1/4000	1/5	0/5	
H6	M	≥8 Yrs	1/40	0/3	0/3	1(1), 2(1)
H9	M	≥8 Yrs	1/40 & 1/4000	2/5	5/5	
H11	M	4-8 Yrs	1/40 & 1/4000	0/4	0/4	1(3)
H15	F	≥8 Yrs	1/40 & 1/4000	0/3	0/3	2(3)
H19	M	≥8 Yrs	1/40	1/5	1/5	
H20	M	≥8 Yrs	1/40 & 1/4000	1/5	5/5	
H23	M	≤ 4 Yrs	1/4000	0/3	1/3	2(2)
H25	M	≥8 Yrs	1/40 & 1/4000	1/3	3/3	2(2)
H28	F	≤ 4 Yrs	1/40 & 1/4000	0/4	3/4	1(2)
H30	F	≥8 Yrs	1/40 & 1/4000	0/1	1/1	4(1)
H33,H43	F	≥8 Yrs	1/40 & 1/4000	0/5	0/5	
H39	M	≥8 Yrs	1/4000	0/5	0/5	
H44	F	4-8 Yrs	1/40 & 1/4000	0/5	1/5	
H45	F	4-8 Yrs	1/40 & 1/4000	0/4	1/4	1(1)
H55	F	≤ 4 Yrs	1/40 & 1/4000	0/3	0/3	1(2)
H56,H60,H65	F	4-8 Yrs	1/40 & 1/4000	0/5	0/5	
H63	F	4-8 Yrs	1/4000	0/5	0/5	
H59	M	4-8 Yrs	1/4000	0/4	0/4	2(2)
H61,H64	F	4-8 Yrs	1/4000	0/4	0/4	2(1),2(1)
H62	F	4-8 Yrs	1/40 & 1/4000	0/4	0/4	1(1)
H66	F	≤ 4 Yrs	1/4000	0/5	1/5	
H67	F	≥8 Yrs	1/40 & 1/4000	0/4	0/4	2(1)
H68	M	4-8 Yrs	1/40 & 1/4000	0/3	0/3	1(2), 1(3)
H69	F	≥8 Yrs	1/40 & 1/4000	0/1	0/1	4(1)

## Discussion

### Overall seroprevalence

We report for the first time evidence for *T. gondii* infection and its seroprevalence, risk factors and bioassay results in Ethiopian camels. An estimated overall seroprevalence of 49.623% by DAT and 40.49% by ELISA tests were detected. Seventy-four percent of seropositive camels (from field samples) had DAT end titer of ≥ 6000. This might indicate actively circulating, recently acquired or recrudescence of previously acquired *T. gondii* infection in camels of the study area due to climatic stress, malnutrition and prevalent diseases like trypanosomosis [36] which reduce the animals’ resistance [37]. Moreover, the seropositive sera that became negative at 1:40 but positive at 1:4000 dilutions (7.34%, 16/218) indicate prozone phenomenon. Presence of viable *T. gondii* cysts in 16.13% (5/31) of camels slaughtered for human consumption is definitive diagnosis of natural *T. gondii* infection and suggests the potential transmission of toxoplasmosis to humans through consumption of raw or undercooked camel meat or offal such as the liver which is commonly consumed by pastoralists. Considering the lower human population density (hence possibly lower domestic cat density), the arid and semi-arid climate of the Fentale district, which is hostile for the survival of oocysts in the environment, and the browsing habit of camel feeding (hence lower risk of acquiring *T. gondii* oocysts from the ground), the high seroprevalence found in the present study was not to our expectation. Recently, a relatively lower seroprevalence of *T. gondii* in goats (15.37%) [25] and in sheep (13.36%) [23] was reported from the same district. However, the high seroprevalence in camels of the current study in our opinion might be a function of cumulative effect of the age of camels [38] related to absence of regular culling programs. Moreover, the migration of camels to midland areas in search of feed, the poor veterinary service, inadequate attention by government, local change in landownership and increased farming [5] - which necessitate cat keeping to control rodents, might have additionally contributed to the high prevalence. The present seroprevalence (48.34%) is in close agreement with the 51.3% and 46% - 54.2% seroprevalence reported from Sudan [39] and Egypt [40,41], respectively. As compared to the present finding, much higher seroprevalence has been reported from Turkey (90.9%) and Sudan (67%) by Utuk et al. [11] and Elamin et al. [8], respectively. Lower seroprevalence was recorded earlier from Iran 3.12% [10], Saudi Arabia 6.5% [42], 16% [43], 13.1% [44], Sudan (20%) [45], United Arab Emirates (22.4%) [46] and Egypt (17.4 - 31.4%) [9,47,48]. The variation in seroprevalence between the present study and aforementioned African and Arabian countries might be due to the difference in density of cats and wild felids, climatic conditions [13], farming and management practices [49], sample size [45], cut-off values and sensitivity difference in the serological tests employed [13,42].

### Risk factors

Assessment of the association of *T. gondii* seroprevalence with potential risk factors was made with the aim of identifying factors relevant for prevention of the disease. Accordingly, using the results of DAT, a multivariable logistic regression model revealed that study PA (*P* = 0.001), exposure to domestic cats (*P* = 0.006) and age of the camels (*P* = 0.033) were found to be independent predictors of *T. gondii* seropositivity. On the other hand, only study PA (*P* = 0.006) and exposure to domestic cats (*P* = 0.011) were found to be independent predictors of *T. gondii* seropositivity using results of ELISA (see Additional file 1). This suggests that DAT is a more preferred test for assessing the risk factors in camels than ELISA. Camels from Ilala PA (82.26%) are 10.8 times more likely to be seropositive than camels of Galcha PA (31.43%) [*P* < 0.001]. The observed difference in seroprevalence across PAs could be due to differences in the age of camels sampled [50], the sample size [44], the degree of environmental contamination [13] and the frequency of exposure to risk factors [51]. The seroprevalence of *T. gondii* infection was higher in camels older than 8 years (OR = 2.49, 95% CI: 1.34, 4.65) as compared to camels ≤ 4 years old. Thus, the probability of *T. gondii* infection in camels increases as the age of the animal increases suggesting postnatal infection. The high seroprevalence in older camels than young camels might be due to the higher likelihood of exposure of older camels to any one of the risk factors to acquire *T. gondii* infection [52]. This finding is in harmony with the studies conducted in Saudi Arabia [8,43] who reported a higher seroprevalence in adult than young camels.

The univariable logistic regression showed that the odds of being infected by *T. gondii* infection was higher in aborted camels than non-aborted camels (OR = 12, 95% CI: 1.25, 114.95; *P* = 0.031) and in areas where the presence of wild felids was reported (OR = 4.28, 95% CI: 2.40, 7.63; *P* < 0.001) than in their absence. However, these variables were not independent predictors of seropositivity in the final logistic regression model (*P* > 0.05). The high seroprevalence in aborted camels is consistent with biological features of *T. gondii* in sheep, goats and pigs. *T. gondii* has been reported as an important cause of abortion in Bacterian camels [53] and in zoo camels in USA [54].

There was no statistically significant sex-linked difference in seroprevalence between male (49.43%; 95% CI: 42.0, 56.86) and female (47.85%; 95% CI: 41.0, 54.65) camels. This is consistent with studies in dromedaries of Sudan [8], Saudi Arabia [43] and bactrian camels of China [55].

### Bioassay

As far as the bioassay result is concerned, tissue cysts were isolated from 16.13% (5∕31) of DAT positive camels. Bradyzoite tissue cysts were detected by microscopy in 22.73% (5/22) of DAT positive mice. This lower rate of detection of cyst by microscopy might be due to the low number of cysts in camel tissue and the low sensitivity of microscopy [13]. Cyst count has also been reported to vary depending on the virulence of *T. gondii* strain and number of passages in the mice [56]. Previously, viable tissue cysts have been isolated in cats from naturally infected camels in Saudi Arabia [16]. It has also been reported that the success of isolation of *T. gondii* cysts as well as the number of tissue cysts produced vary considerably with the intermediate hosts, being higher in sheep, goats and pigs [49,57]. The number of isolated tissue cysts ranged from 25 to 88 per brain of mice, which is low compared to the number of cysts/mouse brain isolated from sheep and goats of the same district (Gebremedhin et al., unpublished). More sensitive methods like PCR might detect the DNA of tissue cysts in the brain of seropositive mice presently reported perhaps as false negative due to the small volume of brain homogenate examined (50 μl), lower sensitivity of microscopy and the possible low burden of viable cysts in tissue of camels [13]. Even though detection of brain cysts was not possible, manifestations of tachypnoea and neurological signs by a mouse on day 20 post-inoculation might indicate the more virulent nature of the strain [13]. The death of mice in less than 3 days following i.p. inoculation was attributed to septic peritonitis that resulted from bacterial contamination of isolates or unintended puncturing of the intestine or other vital organs [34].

### Comparison of seroprevalence results of DAT and ELISA

Comparison of the diagnosis of *T. gondii* infection of camels by DAT and ELISA showed moderate agreement (Kappa = 0.41, *P* < 0.0001). We think that the discrepancies between the results of DAT and ELISA might be ascribed to the difference in the sensitivity and specificity of the tests in camel. DAT is more sensitive than ELISA which is in accordance with findings of Dubey [58] and Shaapan et al. [59] who reported DAT as the most sensitive test among all serological tests. It has also been reported that test performances may vary when applied on different animal species [60]. For example, as opposed to our finding, Zhu et al. [61] from Beijing, China, reported significantly high *T. gondii* seroprevalence by ELISA (34.7%) as compared to DAT (23.1%, cut-off:1:25) in dogs (high degree of agreement, Kappa = 0.644), while no significant difference between the two tests was reported in the case of cats. The P30 ELISA test we used in the current study employs non-species specific conjugate and was recommended by the manufacturer to detect anti-*T. gondii* antibodies in ruminant, cat or pig sera, plasma or meat juice. However, literature with regards to validation of this kit for camels was not accessible; consequently comparison of our results with others couldn’t be made possible. Nevertheless, isolation of viable cysts from four of five camels positive by ELISA (out of ten camel tissues positive by bioassay using DAT, seven were also ELISA positive) coupled with the relatively higher specificity (79.83%) and PPV (73.74%) of ELISA in the present study, suggests the usefulness of the P30 ELISA test in epidemiological studies of camel toxoplasmosis particularly among highly disease prevalent populations and in instances of abortion storms [62]. Although in the present study DAT is more sensitive than ELISA, the subjective nature and longer waiting time before reading the results might limit widespread and large scale usage of DAT for epidemiological studies [61].

### Camel owners’ food habit and awareness about toxoplasmosis

From the questionnaire data it was evident that all of the respondents were uneducated and have a very poor knowledge of *T. gondii* and its mode of transmission between animals, to humans and the role of felids in the epidemiology of the disease. The lack of knowledge about the disease is one of the main risk factors exposing individuals to the disease, as all of them drink raw camel milk and 32.3% responded that they eat either cooked or raw camel meat.

Human cases of toxoplasmosis have previously been linked directly to the consumption of raw goat’s milk [63] and camel milk [8]. Recently, *T. gondii* tachyzoites have been detected from milk of naturally infected camels in Iran using ELISA, PCR, cell culture and cat bioassay [10] and from experimentally infected camels in Sudan [64]. The high seropositivity and moderately high rate of isolation of viable tissue cysts from edible organs in the current study might be of great public health significance. This is due to the deep rooted habit of consumption of raw camel milk, offal (liver) and to a lesser extent, meat by the pastoral communities of the study area. Moreover, the absence of precautions in handling aborted materials and poor knowledge of pastoralists about toxoplasmosis and the role of cats in the transmission of the disease are additional suggestive evidences for the spread of toxoplasmosis to humans and among camels in the study district. In the absence of a vaccine for camels and the difficulty in changing the pastoral extensive livestock management system to intensive management to reduce the prevalence of the disease, prevention of the disease through education of pastoralists remains a better alternative and perhaps a more economically feasible option to combat the disease.

The limitations of the current study include interviewee recall bias, the relatively small number of camels considered for bioassay and the inability to genotype the isolates due to a shortage of resources. Further studies to improve the sensitivity and specificity of ELISA for diagnosis of toxoplasmosis in camels are required.

## Conclusions

*T. gondii* infection of camels in the study district is widespread. Age, presence of domestic cats and study PA are independent predictors of *T. gondii* seropositivity. Isolation of viable parasites from edible tissues of camels and the very poor knowledge of pastoralists about toxoplasmosis, suggest that people are at a higher risk of acquiring *T. gondii* infection. Therefore, prevention of toxoplasmosis through biosecurity measures, education of pastoralists about the identified risks and further investigation to unravel the economic and public health implications of the high prevalence of *T. gondii* in the area deserves consideration.

## Additional file

Additional file 1: Table S1. containing results of analysis based on ELISA for animal level seroprevalence, **Table S2.** herd level seroprevalence and **Table S3.** logistic regression analysis of predictors of seropositivity.
