# Supplementary material for: First report of Toxoplasma gondii in camels (Camelus dromedarius) in Ethiopia: bioassay and seroepidemiological investigation
Source: BMC Vet Res. 2014 Sep 30;10:222. doi: 10.1186/s12917-014-0222-7 (PMC4189602; doi:10.1186/s12917-014-0222-7)
Supplement: Additional file 1: Table S1. — containing results of analysis based on ELISA for animal level seroprevalence, Table S2. herd level seroprevalence and Table S3. logistic regression analysis of predictors of seropositivity. [file 12917_2014_222_MOESM1_ESM.doc]

Additional file 1

Table S1. *T. gondii* seroprevalence in camels of Fentale district stratified by explanatory variables as detected by ELISA (n=381)

| Variable | Categories | No.  tested | No.  positive | % seroprevalence | Chi-square | P-value |
| --- | --- | --- | --- | --- | --- | --- |
| PA | Galcha | 35 | 10 | 28.57 | 17.7732 | 0.**003** |
|  | Tuttuti | 209 | 70 | 33.49 |  |  |
|  | Saraweba | 38 | 16 | 42.11 |  |  |
|  | Garafafate | 10 | 3 | 30.00 |  |  |
|  | Turo | 29 | 15 | 51.72 |  |  |
|  | Ilala | 60 | 36 | 60.00 |  |  |
|  | Total | 381 | 150 | 39.37 |  |  |
| Sex | Female | 207 | 78 | 37.68 | 0.5416 | 0.462 |
|  | Male | 174 | 72 | 41.38 |  |  |
| Age | ≤4yrs | 107 | 36 | 33.64 | 2.0789 | 0.354 |
|  | 4-8yrs | 182 | 75 | 41.21 |  |  |
|  | >8yrs | 92 | 39 | 42.39 |  |  |
| Ps | Pastoral | 304 | 116 | 38.16 | 0.9260 | 0.336 |
|  | Agro-pastoral | 77 | 34 | 44.16 |  |  |
| Hs | Small | 84 | 31 | 36.90 | 5.9062 | 0.052 |
|  | Medium | 170 | 78 | 45.88 |  |  |
|  | Large | 127 | 41 | 32.28 |  |  |
| Abortion | No | 196 | 70 | 35.71 | 6.5193 | 0.**038** |
|  | Yes | 11 | 8 | 72.73 |  |  |
| Stillbirth | No | 205 | 77 | 37.56 | 0.6700 | 0.715 |
|  | Yes | 2 | 1 | 50 |  |  |
| NNM | No | 1 | 0 | 0.00 | 1.1394 | 0.566 |
|  | Yes | 206 | 78 | 37.86 |  |  |

PA= peasant association, Hs=herd size, Ps= production system, NNM=neonatal mortality, P-values of statistically significant variables were highlighted in bold; No. = number

Table S2. Herd level seroprevalence of *T. gondii* infection in camels of Fentale district as detected by ELISA

| Variable | Categories | No.  tested | No.  positive | % seroprevalence | Chi-square | P-value |
| --- | --- | --- | --- | --- | --- | --- |
| PA | Galcha | 4 | 3 | 75 | 3.5106 | 0.622 |
|  | Tuttuti | 11 | 10 | 90.91 |  |  |
|  | Saraweba | 5 | 5 | 100 |  |  |
|  | Garafafate | 2 | 2 | 100 |  |  |
|  | Turo | 4 | 4 | 100 |  |  |
|  | Ilala | 5 | 5 | 100 |  |  |
| Ps | Pastoral | 20 | 18 | 90 | 1.1759 | 0.278 |
|  | Agro-pastoral | 11 | 11 | 100 |  |  |
| Hs | Small | 15 | 14 | 93.33 | 0.4729 | 0.789 |
|  | Medium | 11 | 10 | 90.91 |  |  |
|  | Large | 5 | 5 | 100 |  |  |
| Abortion | Yes | 8 | 8 | 100 | 0.7436 | 0.389 |
|  | No | 23 | 21 | 91.30 |  |  |
| Stillbirth | Yes | 3 | 3 | 100 | 0.2281 | 0.632 |
|  | No | 28 | 26 | 92.86 |  |  |
| NNM | Yes | 2 | 1 | 50 | 0.7179 | 0.**010** |
|  | No | 29 | 28 | 96.55 |  |  |
| Cat | Yes | 8 | 8 | 100 | 0.7435 | 0.389 |
|  | No | 23 | 21 | 91.30 |  |  |
| Wf | Yes | 25 | 23 | 92 | 0.5131 | 0.474 |
|  | No | 6 | 6 | 100 |  |  |
| River | Yes | 29 | 29 | 100 | - | - |

PA= pastoral association, Hs=herd size, Ps= production system, Wf = presence of wild felids, NNM=neonatal mortality, No. = number

Table S3. Animal level Logistic regression analysis of predictors of *T. gondii* infection in Camels as detected by ELISA (n=381)

| Variable | Category | No. of positive/tested (%) | Univariable | | Multivariable | |
| --- | --- | --- | --- | --- | --- | --- |
|  |  |  | OR (95% CI) | p | OR (95% CI) | P |
| PA | Galcha | 10∕35 (28.57) | 1 |  | 1 | - |
|  | Garafafate | 3∕10 (30.00) | 1.07 (0.41-2.83) | 0.889 | 3.05 (0.88-10.53) | 0.078 |
|  | Tuttuti | 70∕209 (33.49) | 1.26 (0.38-4.17) | 0.706 | 1.30 (0.34-5.04) | 0.703 |
|  | Saraweba | 16∕38 (42.11) | 1.82 (0.36-9.14) | 0.468 | 1.59 (0.47-5.35) | 0.454 |
|  | Turo | 15∕29 (51.72) | 2.68 (0.87-8.29) | 0.087 | 1.02 (0.27-3.86) | 0.973 |
|  | Ilala | 36∕60 (60.00) | 3.75 (1.31-10.77) | 0.**014** | 3.91 (1.47-10.37) | 0.**006** |
| Sex | Female | 78/207 (37.68) | 1 |  |  |  |
|  | Male | 72∕174 (41.38) | 1.17 (0.80-1.70) | 0.419 |  |  |
| Age | ≤4yrs | 36∕107 (33.64) | 1 |  | 1 | - |
|  | 4-8yrs | 75∕182 (41.21) | 1.38 (0.85-2.26) | 0.196 | 1.05 (0.62-1.77) | 0.847 |
|  | >8yrs | 39∕92(42.39) | 1.45 (0.79-2.68) | 0.233 | 1.02(0.56-1.88) | 0.946 |
| Ps ^1^ | Pastoral | 116∕304 (38.16) | 1 |  |  |  |
|  | Agro-pastoral | 34∕77 (44.16) | 1.28 ( 0.54-3.03) | 0.572 |  |  |
| Cat | No | 104∕302 (34.44) | 1 |  | 1 | - |
|  | Yes | 46∕79 (58.23) | 2.65 (1.33-5.29) | **0.006** | 3.48 (1.33-9.13) | 0.**011** |
| Wf^2^ | No | 16∕80 (20.00) | 1 |  | 1 | - |
|  | Yes | 134∕301(44.52) | 3.21 (1.86-5.53) | **<0.001** | 2.04 (0.71-5.85) | 0.183 |
| Abortion | No | 70∕196 (35.71) | 1 |  | 1 | - |
|  | Yes | 8∕11 (90.9172.73) | 4.8 (1.33-17.31) | **0.017** | 3.91 (0.94-2.03) | 0.074 |
| Hs ^3^ | Large | 41/127 (32.28) | 1 |  |  |  |
|  | Small | 31∕84 (36.90) | 1.23 (0.36-4.13) | 0.741 |  |  |
|  | Medium | 78∕170 (45.88) | 1.78 (0.56-5.69) | 0.332 |  |  |

^1^Production system, ^2^ Wild felids, ^3^ Herd size
